# Supplementary material for: Enzymatic Birch reduction via hydrogen atom transfer at [4Fe-4S]-OH2 and [8Fe-9S] clusters
Source: Nat Commun. 2025 Apr 4;16:3236. doi: 10.1038/s41467-025-58418-w (PMC11971306; doi:10.1038/s41467-025-58418-w)
Supplement: Supplementary file 1 — Supplementary Information [file 41467_2025_58418_MOESM1_ESM.pdf]

Supplementary Information

Enzymatic Birch reduction via hydrogen atom transfer at [4Fe-4S]-OH_2 and [8Fe-9S] clusters

Jonathan Fuchs[1], Unai Fernandez Arevalo[2], Ulrike Demmer[3], Eduardo Díaz[2], G. Matthias Ullmann[3], Antonio Pierik[4], Ulrich Ermler[5], Matthias Boll[1*]

Supplementary Tables 1-4

Supplementary Figures 1-13

Supplementary Methods

Supplementary References

Supplementary Table 1. X-ray structural analysis of data sets of three different states.

**Data set**	**BzdNO-benzoyl-CoA**	**BzdNO-dienoyl-CoA**	**BzdNO (partially CoA free)**
**Data collection**			
Wavelength [Å]	1.0	1.0	1.0
Space group	C2	C2	C2
Resolution [Å]	50.0-1.35 (1.35-1.45)	50.0-1.45 (1.45-1.55)	50.0-1.6 (1.6-1.7)
Cell axes [Å] [°]	208.9, 102.8, 86.8 102.5	208.2, 102.8, 86.8 102.4	208.2, 102.7, 86.9 102.5
Mol. asym. unit	2	2	2
Completeness [%]	99.3 (99.2)	98.5 (96.1)	86.1 (41.9)
R_{sym} [%]	5.2 (101.8)	6.1 (110.6)	6.4 (47.7)
I/σ_I	11.1 (1.4)	10.3 (1.3)	10.5 (1.6)
Redundancy	3.4 (3.1)	3.4 (3.1)	3.3 (2.0)
CC	99.9 (47.3)	99.8 (56.2)	99.6 (73.5)
**Refinement**			
No. of atoms polypeptide, ligand, solvent	13265, 220, 874	13268, 358, 910	13200, 204, 796
Resolution [Å]	1.35 - 50.0	1.45 - 50.0	1.6 - 50.0
Reflections	388608	309764	201899
$R_{working}$, R_{free} [%]	20.0, 21.8	18.1, 19.9	18.3, 21.1
$B_{average}$ [A^2] polypeptide, ligand, solvent	25.1, 23.6, 27.4	25.4, 22.9, 28.8	30.8, 34.1, 32.2
rmsd: bond length [Å] bond angles [°]	0.008 1.0	0.009 1.1	0.015 1.4
Clash score	4.2	3.8	4.2
Ramachandran favored [%] outliers [%]	97.6 0.3	97.6 0.3	97.6 0.3

Supplementary Table 2. Atoms of the quantum region.

Residue	Atom Name	Atom in QM Calculations
TRP46	CB	0-C
TRP46	HB2	1-H
TRP46	HB3	2-H
TRP46	CG	3-C
TRP46	CD1	4-C
TRP46	HD1	5-H
TRP46	NE1	6-N
TRP46	HE1	7-H
TRP46	CE2	8-C
TRP46	CD2	9-C
TRP46	CE3	10-C
TRP46	HE3	11-H
TRP46	CZ3	12-C
TRP46	HZ3	13-H
TRP46	CZ2	14-C
TRP46	HZ2	15-H
TRP46	CH2	16-C
TRP46	HH2	17-H
GLU65	CG	18-C
GLU65	HG2	19-H
GLU65	HG3	20-H
GLU65	CD	21-C
GLU65	OE1	22-O
GLU65	OE2	23-O
GLU65	HE1	24-H
CYS95	CB	25-C
CYS95	HB2	26-H
CYS95	HB3	27-H
CYS95	SG	28-S
CYS128	CB	29-C
CYS128	HB2	30-H
CYS128	HB3	31-H
CYS128	SG	32-S
HIS131	CB	33-C
HIS131	HB2	34-H
HIS131	HB3	35-H
HIS131	CD2	36-C
HIS131	HD2	37-H
HIS131	CG	38-C
HIS131	NE2	39-N
HIS131	HE2	40-H
HIS131	ND1	41-N
HIS131	CE1	42-C
HIS131	HE1	43-H
CYS380	CB	44-C
CYS380	HB2	45-H
CYS380	HB3	46-H
CYS380	SG	47-S
COA	C2P	48-C
COA	H2P1	49-H
COA	H2P2	50-H
COA	S1P	51-S
BEZ	C	52-C
BEZ	O1	53-O
BEZ	C1	54-C
BEZ	C2	55-C
BEZ	H2	56-H
BEZ	C3	57-C
BEZ	H3	58-H
BEZ	C4	59-C
BEZ	H4	60-H
BEZ	C5	61-C
BEZ	H5	62-H
BEZ	C6	63-C
BEZ	H6	64-H
SF4	FE1	65-Fe
SF4	FE2	66-Fe
SF4	FE3	67-Fe
SF4	FE4	68-Fe
SF4	S1	69-S
SF4	S2	70-S
SF4	S3	71-S
SF4	S4	72-S
WAT	O	73-O
WAT	H1	74-H
WAT	H2	75-H
Link atom		76-H
Link atom		77-H
Link atom		78-H
Link atom		79-H
Link atom		80-H
Link atom		81-H
Link atom		82-H

Supplementary Table 3: Most probable protonation state of the MM region at pH=7 determined using GMCT. HSP-doubly-protonated histdine, HSD-histdine protonated at ND1, HSE–histdine protonated at NE2. All other residues are set to their standard pronation, i.e. Glu, Asp deprotonated and Lys, Arg protonated).

**Residue**	**Protonation**
HIS73A	HSP
ASP93A	protonated
HIS106A	HSD
HIS137A	HSE
HIS172A	HSE
HIS349A	HSE
HIS375A	HSD
GLU381A	protonated
HIS17B	HSE
HIS58B	HSP
HIS67B	HSD
HIS113B	HSE
GLU128B	protonated
HIS133B	HSP
HIS138B	HSE
HIS210B	HSE
HIS301B	HSP
HIS306B	HSP
ASP328B	protonated
HIS330B	HSE
GLU331B	protonated
HIS340B	HSD

Supplementary Table 4: Relative Energies of the different states depicted in the energy diagram in Figure 6 with the respective Mulliken Spin Populations of the Quantum Centers.

	**Step 1**			**Step 2**		**Step 2 Red**		
	**Start**	**TS**	**End**	**TS**	**End**	**Start**	**TS**	**End**
**rel Energy [kcal/mol]**	**0.0**	**6.8**	**-6.1**	**0.6**	**-7.2**	**0.0**	**2.3**	**-20.6**
**Atom ID**	**Mulliken Spin Distribution**							
A:46:TRP:CB	0.000	0.000	0.000	0.000	0.000	0.000	0.000	0.000
A:46:TRP:HB2	0.000	0.000	0.000	0.000	0.000	0.000	0.000	0.000
A:46:TRP:HB3	0.000	0.000	0.000	0.000	0.000	0.000	0.000	0.000
A:46:TRP:CG	0.000	0.001	0.000	0.000	0.000	0.000	0.000	0.000
A:46:TRP:CD1	0.000	0.000	0.000	0.000	0.000	0.000	0.000	0.000
A:46:TRP:HD1	0.000	0.000	0.000	0.000	0.000	0.000	0.000	0.000
A:46:TRP:NE1	0.000	0.000	0.000	0.000	0.000	0.000	0.000	0.000
A:46:TRP:HE1	0.000	0.000	0.000	0.000	0.000	0.000	0.000	0.000
A:46:TRP:CE2	0.001	0.001	0.000	0.000	0.000	0.000	0.000	0.000
A:46:TRP:CD2	0.000	0.001	0.000	0.000	0.000	0.000	0.000	0.000
A:46:TRP:CE3	0.000	-0.001	0.000	0.000	0.000	0.000	0.000	0.000
A:46:TRP:HE3	0.000	0.000	0.000	0.000	0.000	0.000	0.000	0.000
A:46:TRP:CZ3	0.000	0.000	0.000	0.000	0.000	0.000	0.000	0.000
A:46:TRP:HZ3	0.001	0.001	0.000	0.000	0.000	0.001	0.001	0.001
A:46:TRP:CZ2	0.000	-0.002	-0.001	0.000	0.000	0.000	0.000	0.000
A:46:TRP:HZ2	0.000	0.000	0.000	0.000	0.000	0.000	0.000	0.000
A:46:TRP:CH2	0.000	0.001	0.001	0.000	0.000	0.000	0.000	0.000
A:46:TRP:HH2	0.000	0.000	0.000	0.000	0.000	0.000	0.000	0.000
A:65:GLU:CG	-0.021	-0.002	0.001	0.001	0.001	0.002	0.001	0.000
A:65:GLU:HG2	0.004	0.001	0.001	0.001	0.000	0.001	0.001	0.001
A:65:GLU:HG3	0.022	0.003	0.002	0.001	0.000	0.001	0.001	0.000
A:65:GLU:CD	0.148	0.003	-0.004	-0.003	-0.002	-0.005	-0.004	0.000
A:65:GLU:OE1	0.001	0.001	0.003	0.016	0.000	0.000	0.000	-0.001
A:65:GLU:OE2	0.033	0.004	0.001	0.004	0.000	0.000	0.000	-0.002
A:65:GLU:HE1	0.020	0.007	0.004	0.007	0.000	0.000	0.000	-0.001
A:95:CYS:CB	0.005	0.006	0.006	0.006	0.006	0.003	0.003	0.003
A:95:CYS:HB2	0.005	0.005	0.005	0.005	0.005	0.004	0.004	0.004
A:95:CYS:HB3	0.005	0.005	0.004	0.004	0.004	0.005	0.005	0.005
A:95:CYS:SG	0.200	0.204	0.199	0.197	0.194	0.178	0.177	0.171
A:128:CYS:CB	0.002	0.002	0.000	-0.002	-0.003	0.003	0.003	0.002
A:128:CYS:HB2	-0.008	-0.008	-0.008	-0.008	-0.007	-0.008	-0.008	-0.008
A:128:CYS:HB3	-0.008	-0.008	-0.007	-0.008	-0.008	-0.006	-0.006	-0.006
A:128:CYS:SG	-0.225	-0.230	-0.209	-0.204	-0.208	-0.194	-0.194	-0.191
A:131:HIS:CB	0.000	0.000	0.000	0.000	0.000	0.000	0.000	0.000
A:131:HIS:HB2	0.000	0.000	0.000	0.000	0.000	0.000	0.000	0.000
A:131:HIS:HB3	0.000	0.000	0.000	0.000	0.000	0.000	0.000	0.000
A:131:HIS:CD2	0.001	0.000	-0.001	0.000	0.000	0.000	0.000	0.001
A:131:HIS:HD2	0.000	0.000	-0.001	-0.001	-0.001	-0.001	-0.001	-0.001
A:131:HIS:CG	0.000	0.000	0.001	0.001	0.001	0.001	0.001	0.000
A:131:HIS:NE2	0.000	0.001	-0.001	-0.001	-0.002	-0.001	-0.001	-0.003
A:131:HIS:HE2	-0.001	-0.002	0.000	0.000	0.000	0.000	0.000	0.000
A:131:HIS:ND1	0.000	0.000	0.000	0.000	0.000	0.000	0.000	0.000
A:131:HIS:CE1	0.000	0.000	0.000	0.000	0.000	0.000	0.000	0.000
A:131:HIS:HE1	0.000	0.000	0.000	0.000	0.000	0.000	0.000	0.000
A:380:CYS:CB	0.002	0.002	0.002	0.002	0.002	0.001	0.001	0.001
A:380:CYS:HB2	0.002	0.002	0.002	0.002	0.002	0.002	0.002	0.002
A:380:CYS:HB3	0.004	0.004	0.004	0.004	0.004	0.004	0.003	0.003
A:380:CYS:SG	0.152	0.154	0.151	0.153	0.150	0.142	0.139	0.128
C:1:COA:C2P	-0.001	-0.002	-0.002	-0.001	0.000	0.000	0.000	0.000
C:1:COA:H2P1	0.002	0.003	0.003	0.001	0.000	0.000	0.000	0.000
C:1:COA:H2P2	0.000	0.000	0.000	0.000	0.000	0.000	0.000	0.000
C:1:COA:S1P	0.026	0.046	0.045	0.020	0.000	0.000	0.000	0.000

C:2:BEZ:C	0.106	0.072	-0.002	-0.003	0.000	0.000	0.000	0.000
C:2:BEZ:O1	0.083	0.100	0.052	0.011	0.000	0.000	0.000	0.000
C:2:BEZ:C1	0.092	0.235	0.235	0.073	0.001	0.000	0.000	0.001
C:2:BEZ:C2	0.063	0.020	-0.055	-0.007	-0.004	-0.002	-0.003	-0.005
C:2:BEZ:H2	-0.001	0.000	0.004	0.001	0.000	0.001	0.001	0.001
C:2:BEZ:C3	0.004	0.139	0.196	0.067	0.003	0.002	0.002	0.002
C:2:BEZ:H3	-0.010	-0.017	-0.014	-0.006	-0.001	-0.003	-0.003	-0.004
C:2:BEZ:C4	0.145	0.096	-0.028	-0.004	0.000	0.001	0.002	0.000
C:2:BEZ:H4	-0.010	0.021	0.028	0.012	0.000	0.000	0.000	0.000
C:2:BEZ:C5	-0.035	0.061	0.148	0.045	0.001	-0.001	-0.001	0.001
C:2:BEZ:H5	0.001	-0.006	-0.008	0.000	0.000	0.000	0.000	0.000
C:2:BEZ:C6	0.083	0.011	-0.061	-0.019	-0.002	0.000	0.000	-0.003
C:2:BEZ:H6	-0.006	-0.001	0.003	0.001	0.000	0.000	0.000	0.000
C:3:SF4:FE1	-3.056	-3.045	-2.793	-2.549	-2.326	-3.062	-3.065	-3.077
C:3:SF4:FE2	-3.067	-3.152	-3.032	-2.858	-2.781	-3.238	-3.234	-3.218
C:3:SF4:FE3	3.090	3.076	3.078	3.044	3.020	3.132	3.131	3.132
C:3:SF4:FE4	3.154	3.149	3.080	3.017	2.979	3.154	3.156	3.183
C:3:SF4:S1	0.088	0.078	0.086	0.085	0.085	0.096	0.099	0.099
C:3:SF4:S2	0.056	0.050	0.038	0.028	0.023	0.054	0.055	0.056
C:3:SF4:S3	-0.103	-0.107	-0.055	-0.019	-0.004	-0.100	-0.099	-0.088
C:3:SF4:S4	-0.060	-0.068	-0.040	-0.008	0.015	-0.064	-0.063	-0.050
C:4:WAT:O	-0.003	0.018	-0.092	-0.116	-0.150	-0.098	-0.104	-0.139
C:4:WAT:H1	0.001	-0.003	-0.006	-0.007	-0.007	-0.004	-0.005	-0.004
C:4:WAT:H2	0.006	0.065	0.029	0.007	-0.001	-0.001	-0.002	-0.002
CAP-H	0.000	0.000	0.000	0.000	0.000	0.000	0.000	0.000
CAP-H	0.004	0.000	0.000	0.000	0.000	0.000	0.000	0.000
CAP-H	0.005	0.005	0.005	0.006	0.006	0.006	0.006	0.005
CAP-H	-0.006	-0.006	-0.004	-0.003	-0.003	-0.005	-0.005	-0.005
CAP-H	0.000	0.000	0.000	0.000	0.000	0.000	0.000	0.000
CAP-H	0.005	0.005	0.006	0.006	0.006	0.005	0.005	0.004
CAP-H	0.000	0.001	0.001	0.000	0.000	0.000	0.000	0.000

a

b

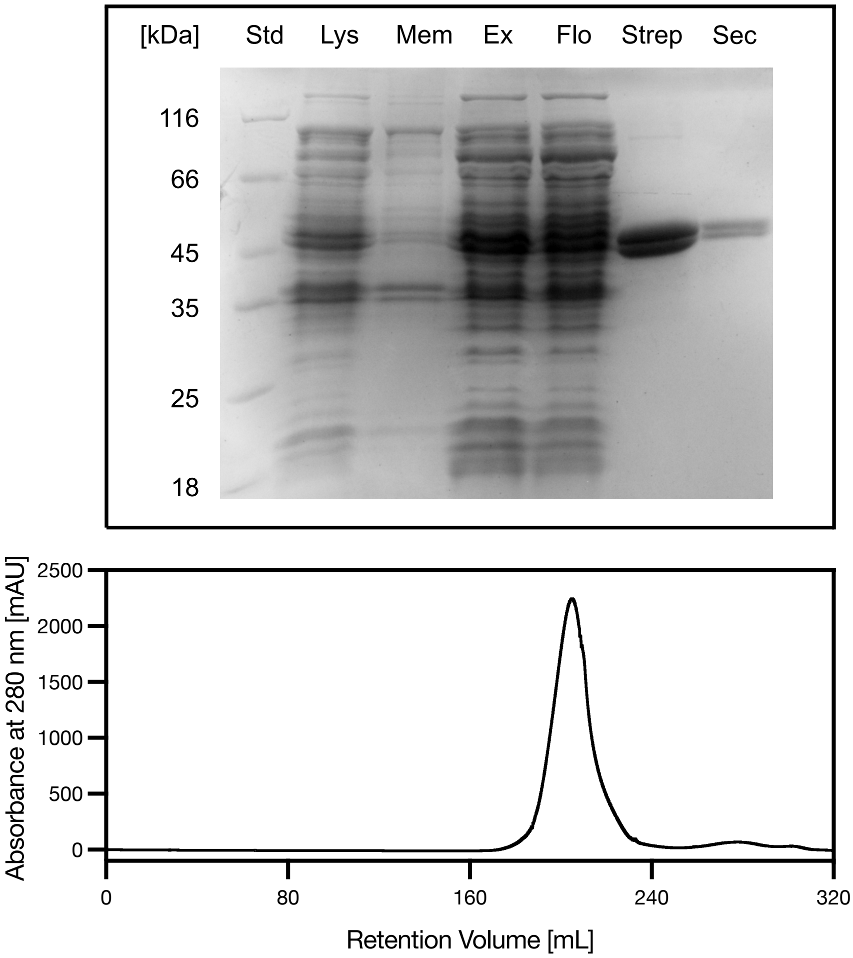

Supplementary Figure 1. Enrichment of BzdNO. (**a**) SDS-PAGE analysis of protein fractions obtained during the enrichment of BzdNO after heterologous production; (**b**) chromatogram obtained during BzdNO enrichment by preparative size exclusion chromatography using a HiLoad 26/600 Superdex 200 pg resin. Std, molecular mass standard; Lys, cell lysate; Mem, membrane fraction after ultracentrifugation; Ex, soluble cell free extract after ultracentrifugation; Flo, flow through after Strep-Tactin® XT 4Flow® affinity chromatography; Strep; Fraction obtained after elution with D-biotin; Sec, fraction obtained after size exclusion chromatography. The enriched two protein bands migrating at around 50 kDa were identified as BzdN and BzdO by mass spectrometric analyses of tryptic peptides, respectively. Source data are provided with this paper.

a

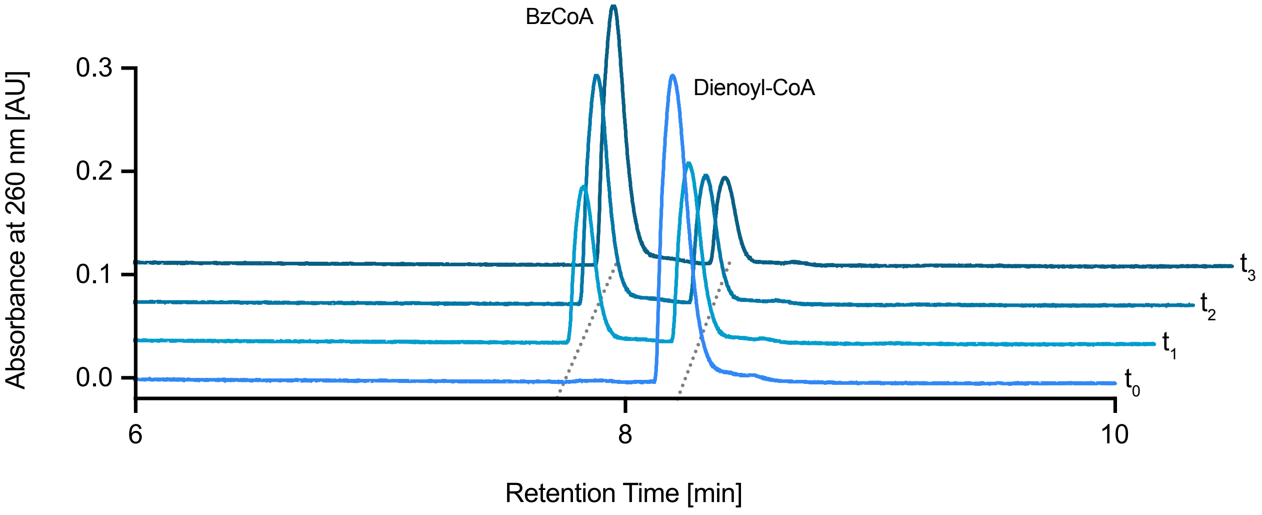

b

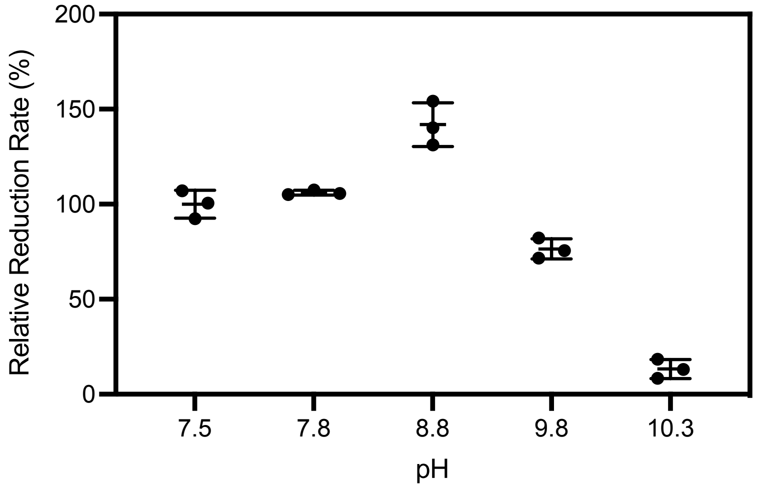

Supplementary Figure 2. Assay and pH dependence of BzdNO activity. (**a**) UPLC-based assay of BzdNO-dependent oxidation of dienoyl-CoA to BzCoA in the presence of the electron acceptor methyl viologen. UPLC-chromatograms of dienoyl-CoA before and 10 min (t_1), 20 min (t_2) and 30 min (t_3) after addition of BzdNO in the presence of 0.5 mM methyl viologen are shown. (**b**) pH dependency of the dienoyl-CoA oxidizing activity of BzdNO (n=3; ± SD). Dienoyl-CoA oxidation was followed by continuous photometric assays monitoring benzyl viologen reduction. Source data are provided with this paper.

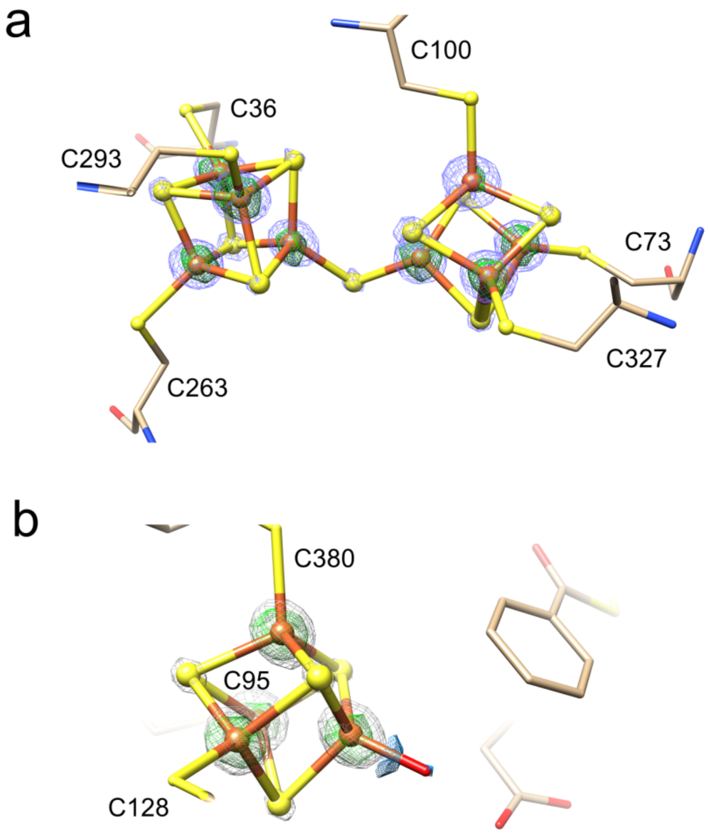

Supplementary Fig. 3. Analysis of the [8Fe-9S] cluster (**a**) and the [4Fe-4S]-OH_2 cluster (**b**) of BzdNO: The anomalous difference electron density (green) for the BzdNO-benzoyl-CoA was given at 12 σ. The omit $2F_{obs}$-F_{calc} electron density was drawn at 5 σ (gray) to distinguish the electron density of Fe, S (Cl). In addition, the omit $2F_{obs}$-F_{calc} electron density around the H_2O ligand of [4Fe-4S]-OH_2 cluster was drawn at 2.5 σ to highlight the separation of the electron density between the Fe and the oxygen. The distance between the centers of Fe and O is 1.89 ± 0.1 Å. In comparison the Fe-S bond would be ca. 2.3 Å.

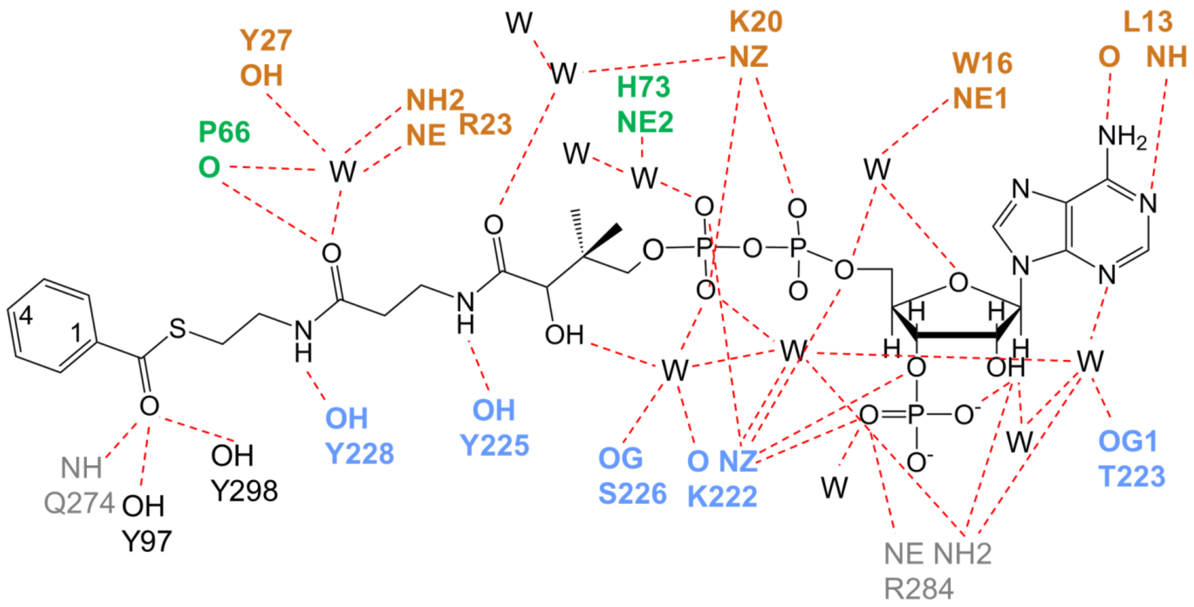

Supplementary Fig. 4. Scheme of substrate binding showing the amino acids in contact with BzCoA. Hydrogen bonds are drawn as red dashed lines; hydrophobic contact as black dotted lines. The CoA binding site is embedded into a channel primarily formed by helices 15:32 (orange), 64:70 (green), 220:234 (blue) and 281:288 (gray) as well as neighbored loops.

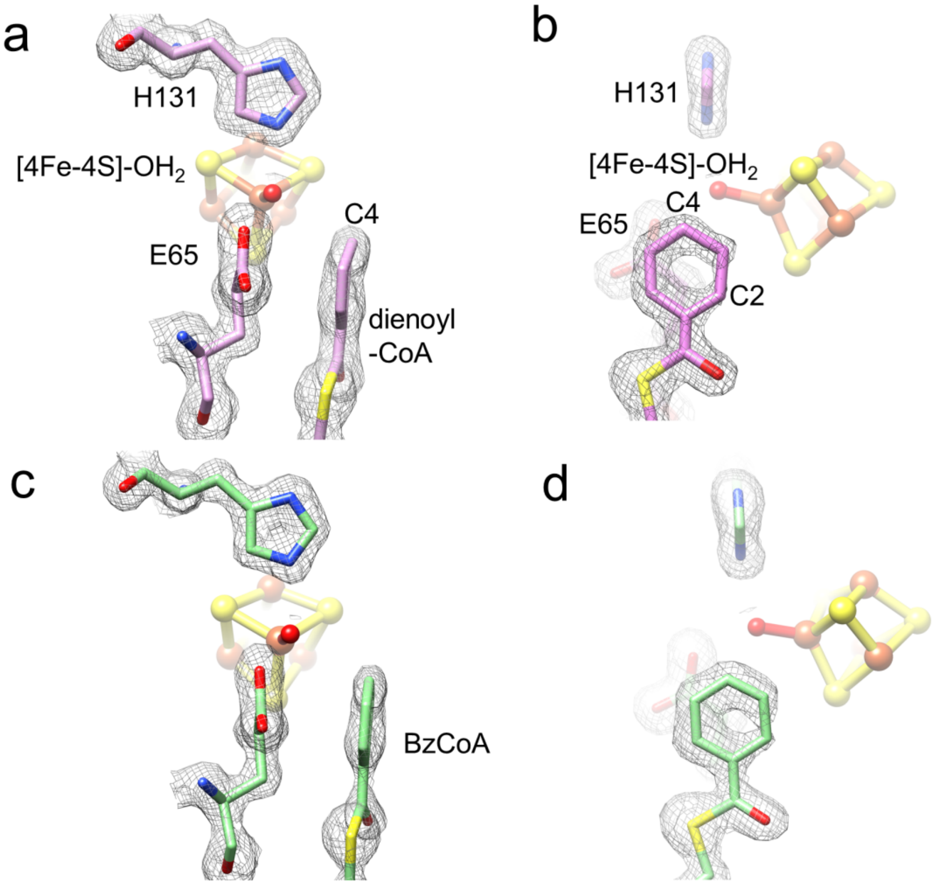

Supplementary Fig. 5. The conformation of the six-membered ring of dienoyl- and benzoyl-CoA and their position relative to the [4Fe-4S]-OH_2 cluster and His131. Upper panel: The BzdNO-dienoyl-CoA complex at 1.45 Å resolution showing a side (**a**) and front view (**b**) of the cyclohexadiene ring (carbon in pink). The contour level of the electron density is 2.1σ. Lower panel: The BzdNO-benzoyl-CoA complex at 1.35 Å resolution with the phenyl ring (carbon in green) in a side (**c**) and front view (**d**). The contour level of the electron density is 2.5σ. Notably, the benzoyl and dienoyl six-membered rings, considered to be occupied by nearly 100%, show higher B factors for C2 (~3-5 $Å^2$) compared with the other ring carbons suggesting a stronger vibration.

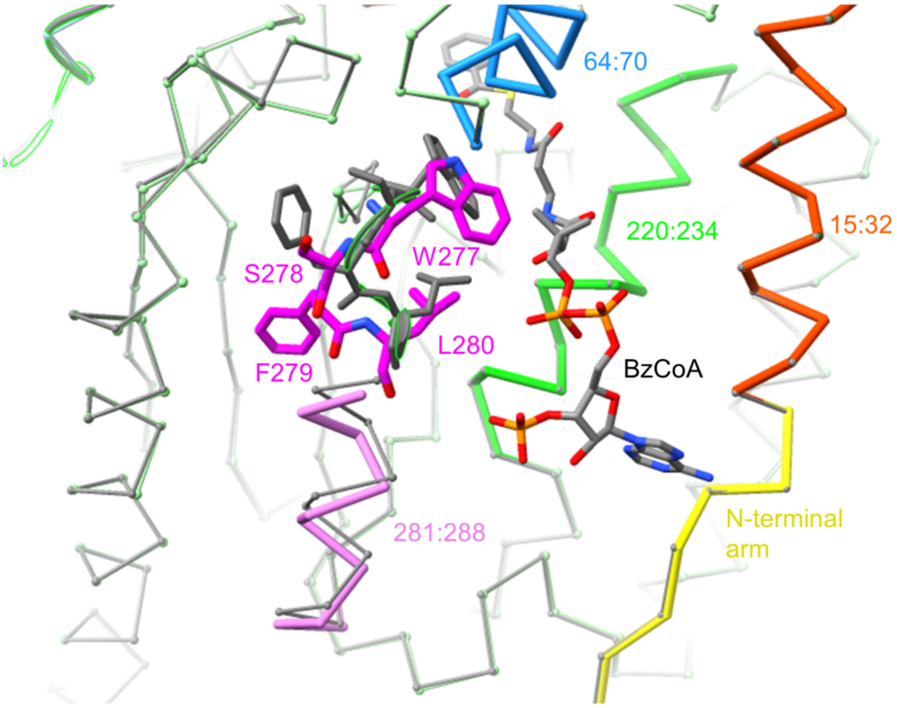

Supplementary Fig. 6. Conformational changes upon substrate binding. Superimposed BzdNO in complex with benzoyl-CoA (gray) and in a partially CoA-free state (orange) are drawn as C_α-plot. In addition, helices 15:32, 64:70, 220:234 and 281:288 involved in forming the CoA binding site are highlighted in orange-red, blue, gray and orange. The major change occurs at helix 281:288 and the preceding loop drawn with side chain. Other CoA binding segments only locally respond to substrate binding by minor side chain rearrangements and B-factor decreases.

.

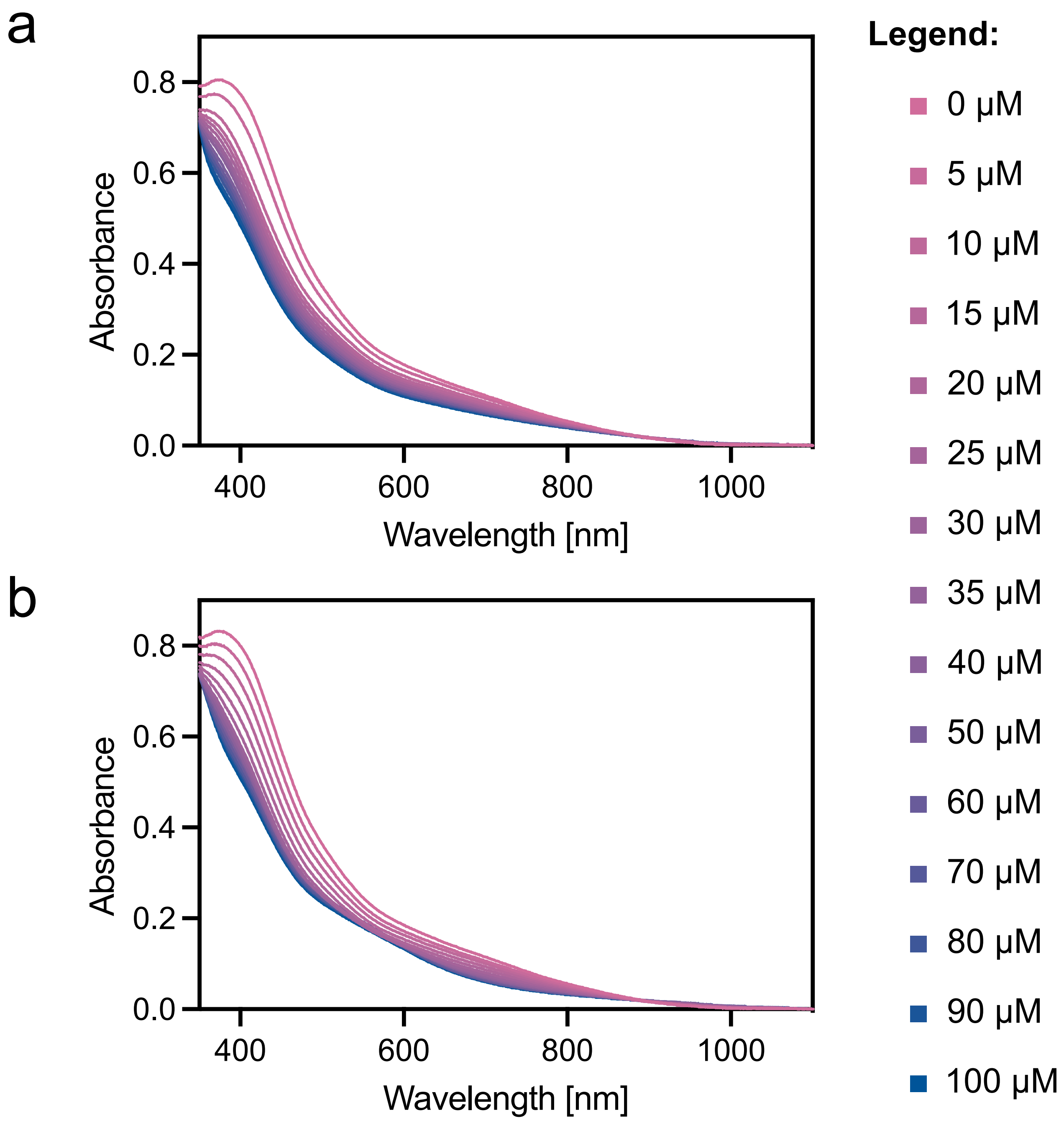

Supplementary Figure 7. UV-vis absorbance spectroscopy of BzdNO upon reduction at pH 9. BzdNO (20 µM) was reduced with 5-100 µM sodium dithionite (**a**) or dienoyl-CoA (**b**) in 100 mM TAPS/KOH pH 9 under anaerobic conditions at 30 °C. The concentration of the reducing agent is given for each spectrum. Source data are provided with this paper.

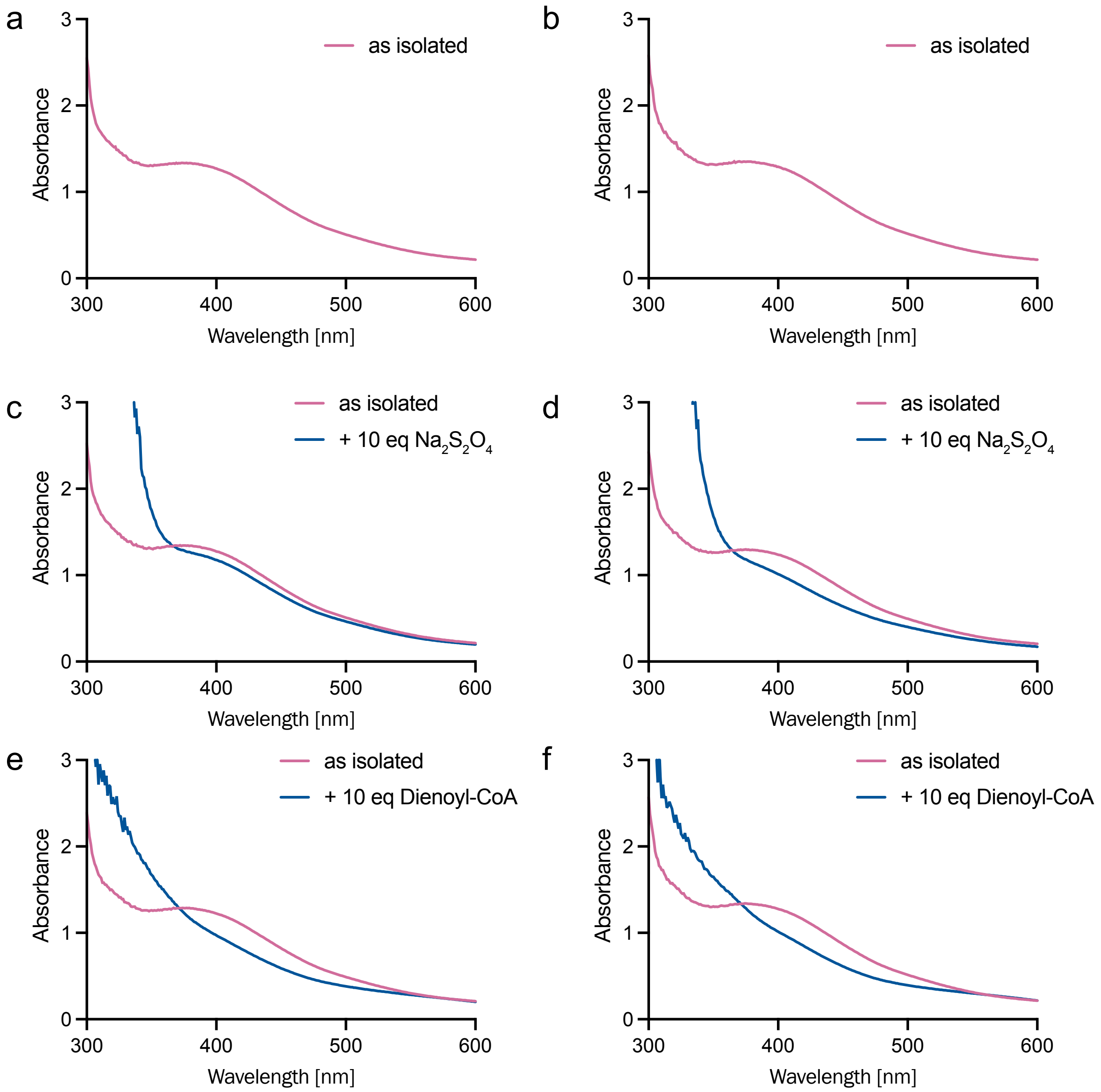

Supplementary Fig. 8. UV-vis absorbance spectra of EPR samples. The absorbance of as-isolated BzdNO (30 µM; **a**/**b**), BzdNO reduced with 10 equivalents sodium dithionite (**c**/**d**) and BzdNO reduced with 10 equivalents dienoyl-CoA (**e**/**f**) is plotted against the wavelength in nm. Samples were prepared in 100 mM MOPS/KOH at pH 7.8 (**a**/**c**/**e**) or 100 mM TAPS/KOH at pH 9.0 (**b**/**d**/**f**) and immediately frozen in EPR tubes upon acquisition of the UV-vis spectra. Source data are provided with this paper.

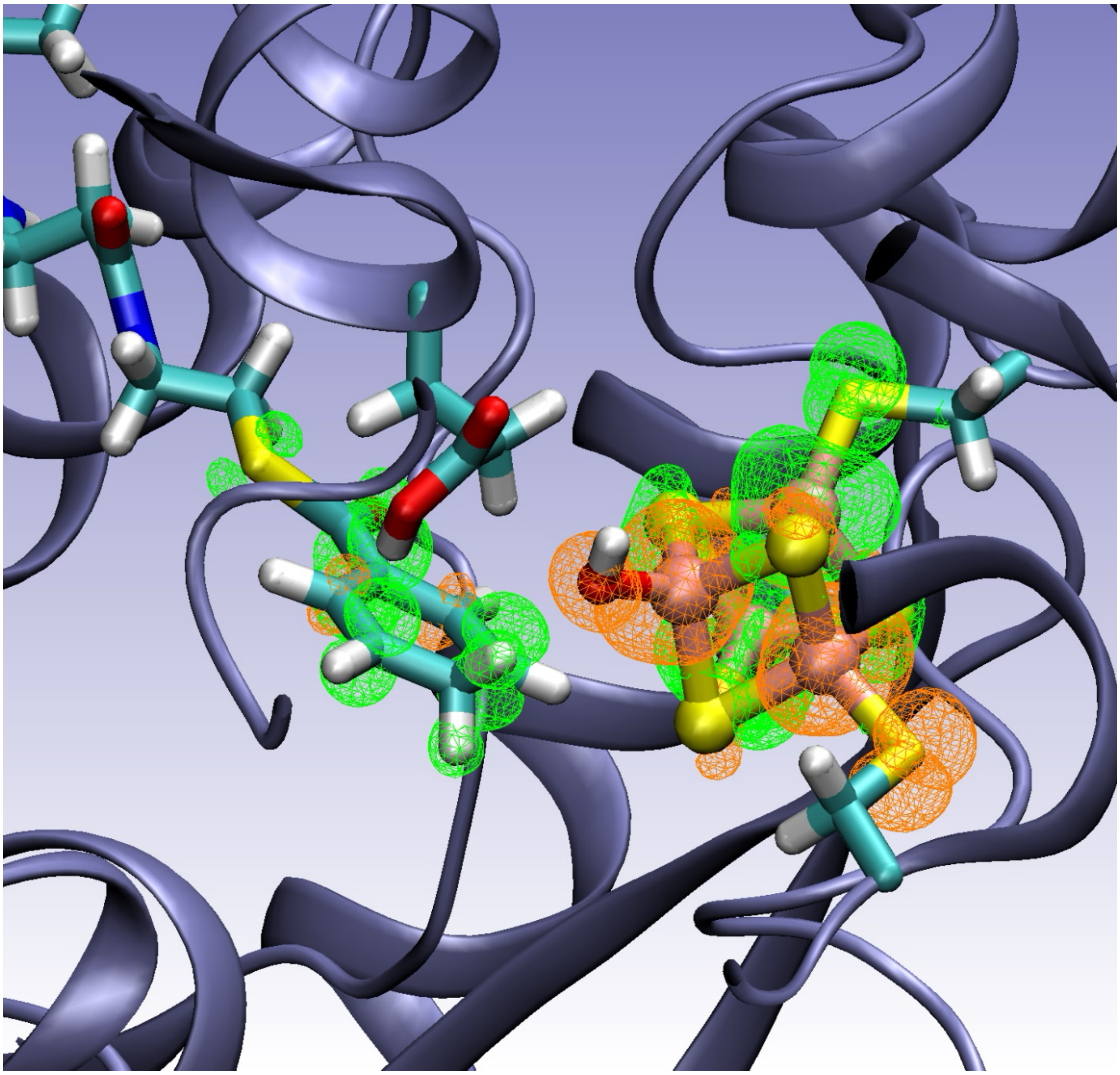

Supplementary Fig. 9: Spin distribution in the reaction intermediate after the first step (contour levels at 0.002 e/A^3). The spin is distributed over the whole aromatic ring and the thio-ester which stabilizes this intermediate. Glu65 is in the correct position to donate the proton for the second step.

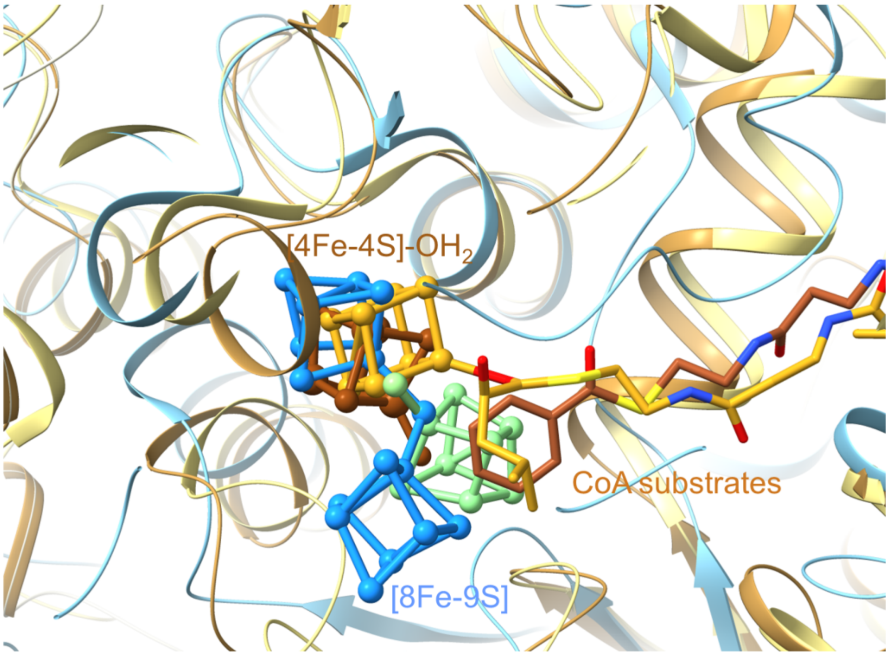

Supplementary Fig. 10. Superposition of the active sites in BzdNO (brown), DCCP (blue), HAD (yellow) and TudS (4-thiouridine-5'-monophosphate desulfidase). The [4Fe-4S]-OH_2 clusters and benzoyl-CoA of BzdNO are drawn in brown, the double-cubane [4Fe-4S] clusters of DCCP in blue, the [4Fe-4S] cluster- hydroxyisocaproyl-CoA adduct in yellow and the [4Fe-4S]-S cluster of the desulfidase in green. The structure of TudS significantly deviates from the other family members and was omitted for clarity.

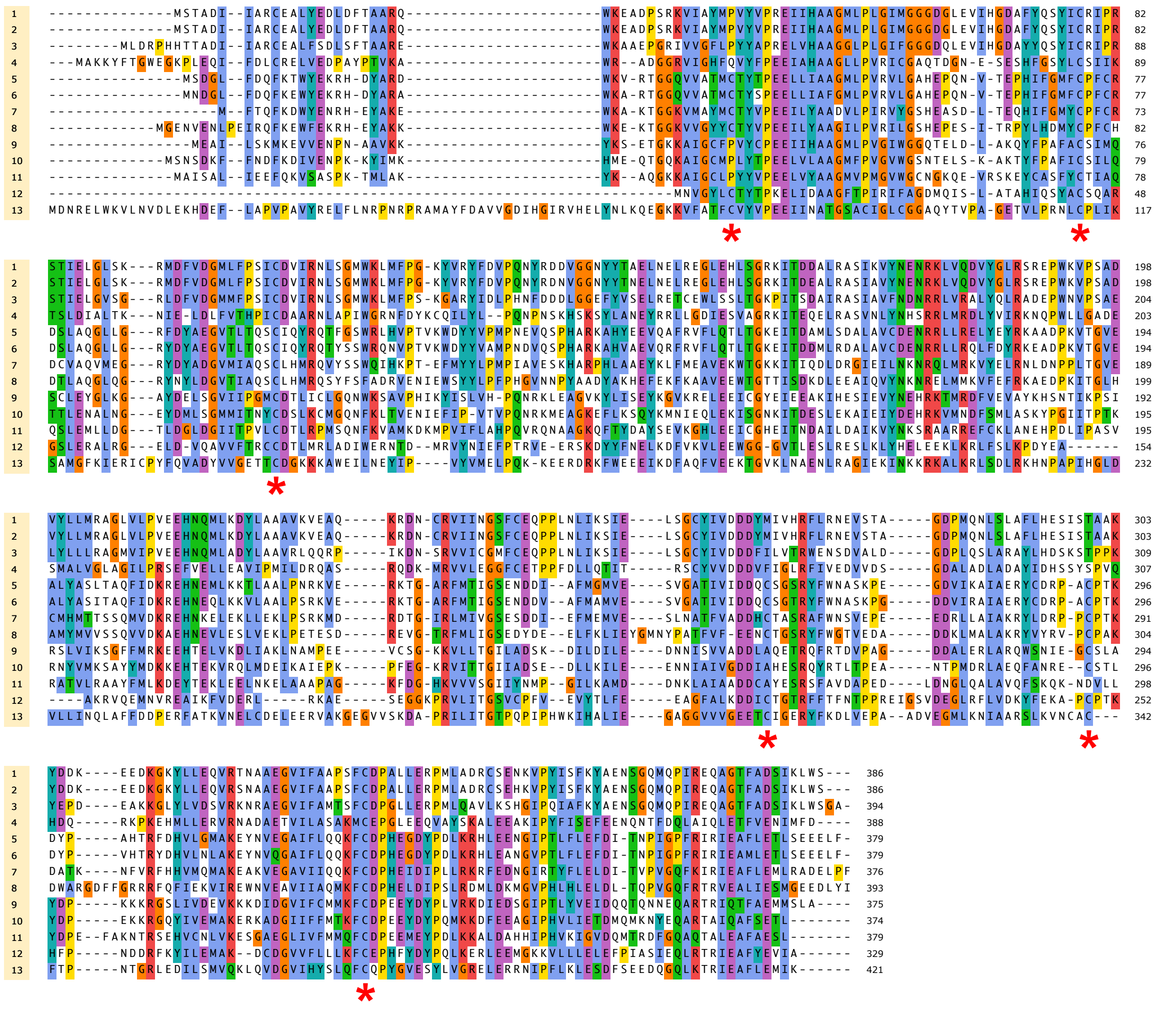

Legend:

1. BcrC *Thauera aromatica* K172 (GenBank CAA12247.1)
2. BcrC *Thauera chlorobenzoica* 3CB-1 (GenBank WP_075148410.1)
3. BcrC *Rhodopseudomonas palustris* (GenBank WP_119017419.1)
4. MbrN *Thauera chlorobenzoica* 3CB-1 (GenBank SEG26980.1)
5. BzdN *Aromatoleum* sp. CIB (GenBank AAQ8806.1)
6. BzdN *Aromatoleum aromaticum* (GenBank WP_011236238.1)
7. ThncrN Enrichment culture N47 (GenBank ADB04315.1)
8. BzdN *Ferroglobus placidus* (GenBank WP_012965685.1)
9. HadC *Clostridioides difficile* ATCC9689 (GenBank AAV40820.1)
10. FdlC *Clostridium sporogenes* (GenBank AAL18811.1)
11. HgdB *Acidaminococcus fermentans* DSM20731 (GenBank P11570.3)
12. HgdB *Archaeoglobus fulgidus* DSM4304 (GenBank AAB89298.1)
13. DCCP *Carboxydothermus hydrogenoformans* (GenBank WP_011343421.1)

Supplementary Fig. 11. Amino acid sequence alignment of BcrC/BzdN and related enzymes. Conserved cysteine residues coordinating the [8Fe-9S] are marked with red asterisks.

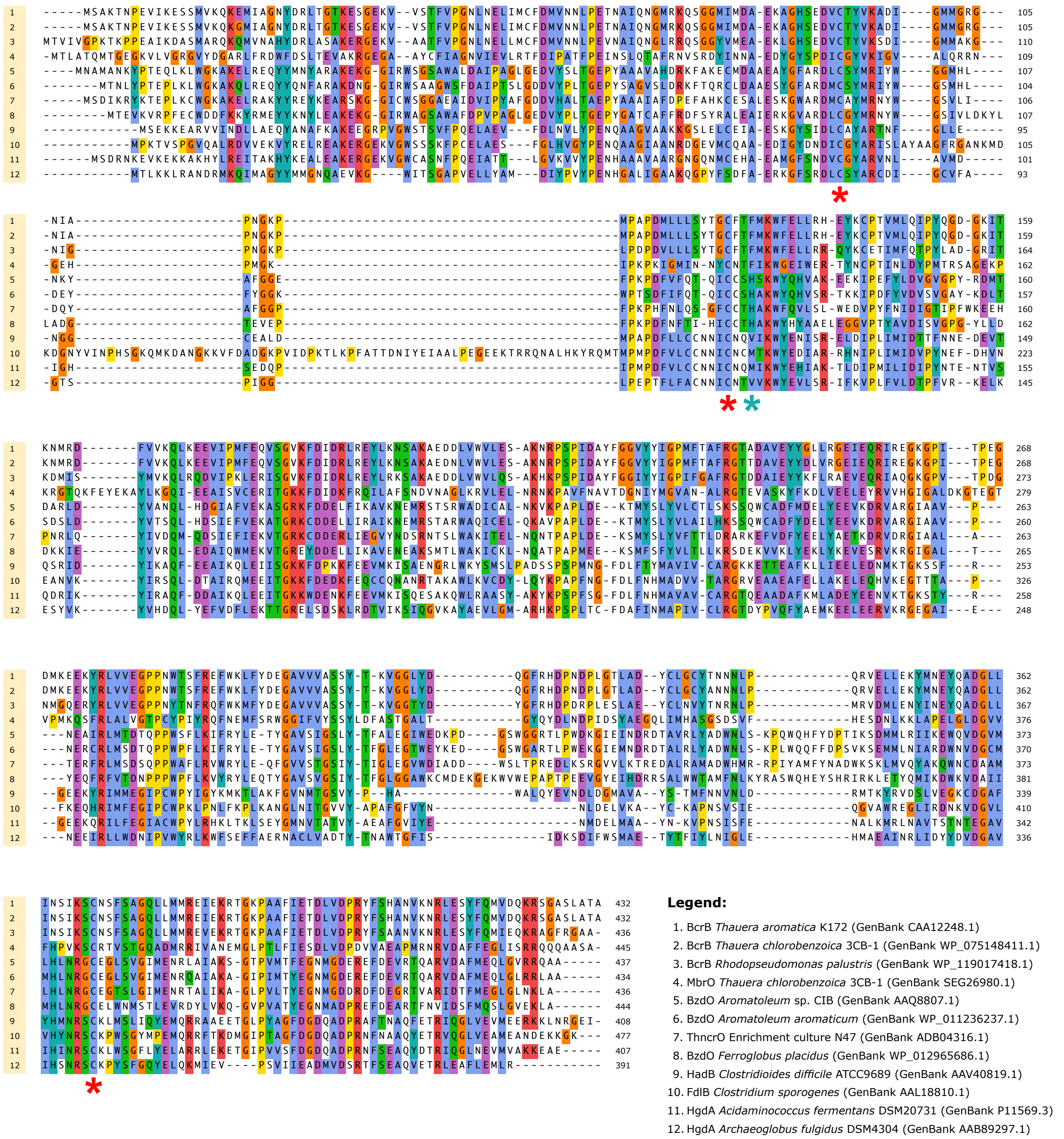

Supplementary Fig. 12. Amino acid sequence alignment of BcrB/BzdO and related enzymes. Conserved cysteine residues coordinating the [4Fe-4S] are marked with red asterisks.

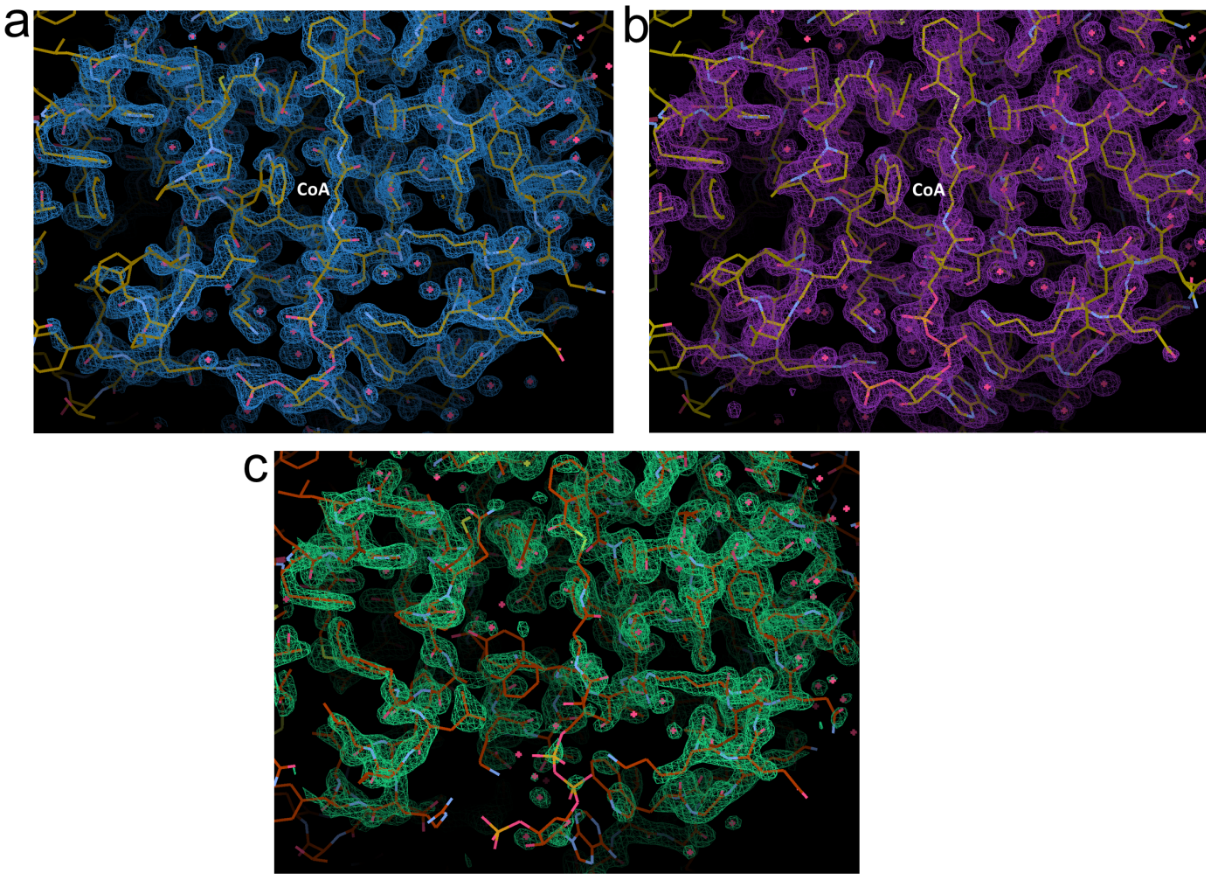

Supplementary Fig. 13. Composite omit electron density maps of the BzdNO-benzoyl-CoA (**a**), BzdNO-dienoyl-CoA (**b**) and BzdNO (partially CoA free) (**c**) structures. The contour levels of the 2mF_{obs}-DF_{calc} maps are 2σ. In all three structures the section around the substrate binding site was chosen.

Supplementary Methods

Details of the QM/MM Setup

The quantum center consists of 74 atoms which are listed in Supplementary Table 2. In the active site, Glu65 was set to be protonated and His131 was set to be singly protonated (with the proton at NE2, since ND1 forms a hydrogen bond with the backbone.). The QM/MM calculations were performed using ORCA. We used unrestricted DFT as QM method, namely we used the BP86 functional with the def2-SVP basis set for the search of the path. Single-point calculations of the end states and the transition states were done using the TPSS functional and the def2-TZVP basis set. The MM energies were calculated using the CHARMM36 force field. To model the QM/MM boundary, a link-atom scheme and electrostatic embedding was used. The QM region was surrounded by a fully flexible MM layer of 8 Å. The [4Fe-4S]-cluster was treated by the broken-symmetry approach. The charge of the system was set to –2 for the first reaction step and to –3 if a re-reduction was considered for the second step. All transition states were searched by the climbing-image nudged-elastic band approach.

For the NEB-calculations, the charges of the titratable residues outside of the quantum center were set to their standard protonation (i.e. Glu, Asp deprotonated, Lys, His, Arg protonated) and the spin of Fe1 and Fe2 in the [4Fe-4S]-cluster were flipped. To check if the results are robust for a different protonation scenario and different spin-flip states, we performed a single point calculation using different charge sets for the MM region and flipping the spin of different states. The protonation set was determined by a Poisson-Boltzmann based calculation of the protonation probability at pH 7 using our program GMCT[1]. This protonation state is given in Supplementary Table 3. Although the detailed energy profile changes for different charge sets, the general reaction scheme suggested in Figure 6 is plausible for the different charge set. The orca output files of the different calculations including the energies, the electronic structure and the spin populations can be found at zenodo (https://doi.org/10.5281/zenodo.14811052).

Supplementary References

1. Ullmann, R. T. & Ullmann, G. M. GMCT : A Monte Carlo simulation package for macromolecular receptors. *J Comput Chem* **33**, 887–900 (2012).
