# Supplementary material for: Enzymatic Birch reduction via hydrogen atom transfer at [4Fe-4S]-OH2 and [8Fe-9S] clusters
Source: Nat Commun. 2025 Apr 4;16:3236. doi: 10.1038/s41467-025-58418-w (PMC11971306; doi:10.1038/s41467-025-58418-w)
Supplement: Supplementary file 2 — Description of Additional Supplementary Files [file 41467_2025_58418_MOESM2_ESM.pdf]

Description of Additional Supplementary Files

File name: Supplementary Movie 1
Description: The video shows the enzymatic aromatic ring reduction at the active site [4Fe-4S]-OH_2 cluster based on QM/MM calculations. The mechanism is shown without re-reduction of the cluster after the initial electron transfer.
